# Atlantic Salmon (*Salmo salar* L.) as a Marine Functional Source of Gamma-Tocopherol

**DOI:** 10.3390/md12125944

**Published:** 2014-12-09

**Authors:** David Menoyo, Carmen Sanz-Bayón, Anna Hesby Nessa, Tuba Esatbeyoglu, Mohammad Faizan, Kathrin Pallauf, Nuria De Diego, Anika Eva Wagner, Ignacio Ipharraguerre, Ingunn Stubhaug, Gerald Rimbach

**Affiliations:** 1 Department of Animal Production, Technical School of Agricultural Engineering, Polytechnic University of Madrid, 28040 Madrid, Spain; E-Mails: david.menoyo@upm.es (D.M.); c.sanz.bayon@gmail.com (C.S.-B.); ndc.agronomos@upm.es (N.D.D.); 2 Skretting Aquaculture Research Centre (ARC), P.O. Box 48, N-4001 Stavanger, Norway; E-Mails: Anna.Hesby.Nessa@skretting.com (A.H.N.); Ingunn.Stubhaug@skretting.com (I.S.); 3 Institute of Human Nutrition and Food Science, University of Kiel, Hermann-Rodewald-Straße 6-8, D-24118 Kiel, Germany; E-Mails: esatbeyoglu@foodsci.uni-kiel.de (T.E.); faizan@foodsci.uni-kiel.de (M.F.); pallauf@foodsci.uni-kiel.de (K.P.); wagner@foodsci.uni-kiel.de (A.E.W.); 4 Lucta, Can Parellada 28, 08170 Montornés del Vallés, Barcelona, Spain; E-Mail: Ignacio.Ipharraguerre@lucta.es

**Keywords:** gamma tocopherol, vitamin E, oily fish, salmon (*Salmo salar*), omega 3 fatty acids, functional foods, cardiovascular disease

## Abstract

Gamma tocopherol (gT) exhibits beneficial cardiovascular effects partly due to its anti-inflammatory activity. Important sources of gT are vegetable oils. However, little is known to what extent gT can be transferred into marine animal species such as Atlantic salmon by feeding. Therefore, in this study we have investigated the transfer of dietary gT into salmon. To this end, fish were fed a diet supplemented with 170 ppm gT for 16 weeks whereby alpha tocopherol levels were adjusted to 190 ppm in this and the control diet. Feeding gT-rich diets resulted in a three-fold increase in gT concentrations in the liver and fillet compared to non-gT-supplemented controls. Tissue alpha tocopherol levels were not decreased indicating no antagonistic interaction between gamma- and alpha tocopherol in salmon. The concentration of total omega 3 fatty acids slightly increased in response to dietary gT. Furthermore, dietary gT significantly decreased malondialdehyde in the fillet, determined as a biomarker of lipid peroxidation. In the liver of gT fed salmon we observed an overall down-regulation of genes involved in lipid homeostasis. Additionally, gT improved the antioxidant capacity by up-regulating *Gpx4a* gene expression in the pyloric caeca. We suggest that Atlantic salmon may provide a marine functional source capable of enriching gT for human consumption.

## 1. Introduction

Cardiovascular disease is the leading cause of mortality in the Western world. There is evidence that the consumption of oily fish may significantly decrease cardiovascular disease risk in humans [1]. Oily fish, such as Atlantic salmon, is an important source of long chain omega 3 fatty acids including eicosapentaenoic acid (EPA; C20:5*n*-3) and docosahexaenoic acid (DHA; C22:6*n*-3) which may exhibit beneficial cardiovascular effects [2,3,4]. Due to their double bonds, both EPA and DHA are prone to lipid peroxidation. Our studies have demonstrated that Vitamin E (alpha tocopherol) significantly prevents the oxidation of long chain PUFAs in oily fish [5]. The antioxidant properties of Vitamin E result from its phenolic hydroxyl group which donates hydrogen to peroxyl radicals, resulting in the formation of relatively stable lipid species [6]. In addition to its antioxidant function, important gene-regulatory properties of Vitamin E have been described [7,8].

Vitamin E comprises a mixture of four tocopherols which are 6-chromanol derivatives connected to an aliphatic side-chain. Individual tocopherols are named according to the position and number of the methyl groups on the phenol ring, with the α-, β-, γ- and δ- vitamins containing three, two, two and one methyl groups, respectively. These structural differences determine the biological activity of tocopherols [6].

Gamma tocopherol (gT) occurs at high concentrations in plant-derived oils including soya- and rapeseed oil [9,10]. Studies suggest that gT exhibits biological functions which clearly distinguishes it from alpha tocopherol [10]. As compared to alpha tocopherol, gT is a more potent scavenger of reactive nitrogen species. In this context, it is postulated that gT acts as a trap for electrophilic nitrogen oxides forming stable carbon-centered adducts through the nucleophilic 5-position [11].

Chronic inflammatory processes are centrally involved in the pathogenesis of cardiovascular diseases. Interestingly, gT, but not alpha tocopherol, inhibited cyclooxygenase 2 in cultured macrophages, thereby mediating potent anti-inflammatory activity [12]. Furthermore, Jiang and co-workers observed a suppression of inducible nitric oxide synthase expression by gT in lipopolysaccharide-challenged macrophages [12]. The anti-inflammatory properties of gT *in vitro* have been subsequently confirmed in corresponding *in vivo* studies in laboratory rodents [13].

Plasma concentrations of gT in humans may be inversely associated with cardiovascular disease risk [10]. Given the unique functional properties and health benefits of gT, it seems plausible to enrich oily fish with gT [14]. While vegetable oils, which are considered an important source of gT, do not contain significant amounts of long chain polyunsaturated fatty acids, oily fish is a source of EPA and DHA. Gamma-tocopherol in combination with the fish oils EPA and DHA may act synergistically as far as their beneficial cardiovascular activities in humans are concerned. In addition, feeding Atlantic salmon with gT might improve fish antioxidant status thereby preventing the oxidation of EPA and DHA. This could be a feasible nutritional strategy for the salmon feed industry to maintain high tissue EPA and DHA levels in a context of scarce fish oil supply as a raw material for feed production.

In this study we have aimed to investigate if dietary gT affects tocopherol levels, antioxidant status and fatty acid composition of Atlantic salmon as an important source of EPA and DHA and as a potential functional source of gT for human nutrition.

To this end we have measured malondialdehyde (MDA) concentrations as a biomarker of lipid peroxidation and sensory quality [15] and carried out fatty acid gas chromatography analyses, antioxidant enzyme activity measurements and gene expression analyses of genes involved in fatty acid transport, synthesis and metabolism.

## 2. Results

### 2.1. Fish Performance

The growth of the fish was divided into two periods, due to detection of the parasite Ichthyobodo in the fish after 12 weeks of feeding. Fish were then treated with formalin (30 min bath in 1:4000 ppm of 35% formalin) and fed for another 4 weeks prior to sampling. There were no differences in specific growth rate (SGR) or feed conversion ratio (FCR) before and after the formalin treatment. In the first growth period, the fish had an average SGR of 1.19 ± 0.04 and a FCR of 0.81 ± 0.04. In the second period after the formalin treatment, the SGR was 1.55 ± 0.07 and the FCR 0.74 ± 0.02.

### 2.2. Tissue Levels of Tocopherols, Malondialdehyde, Antioxidant Enzymes and Fatty Acid Composition

In order to evaluate the transfer of Vitamin E from the diet into the tissues of Atlantic salmon we determined the gamma and alpha tocopherol concentrations in fish fillet and liver. As summarized in Table 1, feeding the gT enriched diet resulted in a three-fold increase of gT concentrations in both fillet and liver (*p* < 0.0001). However, alpha tocopherol levels in fillet and liver were similar between the groups indicating no antagonistic interaction between gamma and alpha tocopherol.

Most methods for analyzing lipid peroxidation in animal tissues lack specificity and sensitivity [16]. However, MDA is an important biomarker of lipid peroxidation and may also partly reflect the sensory quality of fish [15]. Using a validated HPLC method [17] to measure MDA, we found that MDA concentrations were significantly decreased by 25% in the fillet of gT-fed salmon as compared to the controls (3.3 ± 0.2 mmol·MDA/kg fillet in the control *vs.* 2.5 ± 0.2 mmol·MDA/kg fillet in gT-fed salmon, *p* < 0.02).

**Table 1:** Alpha and gamma tocopherol concentrations (μmol/kg) in fillet and liver of Atlantic salmon fed either the control diet (C) or a diet enriched with gamma tocopherol (gT) ^a^.

Tissue	C	gT	*p*
Fillet			
α-tocopherol	36.7 ± 3.2	45.3 ± 4.4	0.86
γ-tocopherol	5.4 ± 0.3	16.3 ± 1.1	<0.0001
Liver			
α-tocopherol	912 ± 69	937 ± 131	0.15
γ-tocopherol	34.7 ± 2.4	112.1 ± 9.9	<0.0001

^a^ Values are means ± SE (*n* = 8).

Liver superoxide dismutase and catalase activities were lower (*p* < 0.05) in gT-supplemented salmon as compared to the controls (Table 2). There were no differences in hepatic glutathione concentrations between the groups (Table 2).

**Table 2:** Hepatic catalase (CAT) and superoxide dismutase (SOD) activities as well as glutathione concentrations (GSH) in Atlantic salmon fed either the control diet (C) or a diet enriched with gamma tocopherol (gT) ^a^.

Biomarker	C		gT	*p*
SOD (U/mg protein)	24.6	±2.6	18.0 ± 1.2	0.04
CAT (U/mg protein)	46.8	±3.3	35.8 ± 2.1	0.01
GSH (µmol/g)	2.1	±0.1	1.9 ± 0.1	0.22

^a^ Values are means ± SE (*n* = 8).

The fatty acid composition of the fish fillet reflected, by and large, the fatty acid composition of the diet. Interestingly, gT fed salmon exhibited a slightly higher *n*-3/*n*-6 ratio (*p* = 0.01) in comparison to the control animals (Table 3). The increase in the *n*-3/*n*-6 ratio was related to an increase in *n*-3 fatty acids since the amount of *n*-6 fatty acids remained unchanged. As far as *n*-3 fatty acids are concerned, gT supplementation somewhat increased C18:3*n*-3 in fish fillet (*p* = 0.02). However, the concentration of long chain PUFAs including EPA and DHA was not significantly different between the control and gT-fed fish.

The liver fatty acid composition also reflected the dietary profile (Table 4). Gamma tocopherol promoted the accumulation of C18:0 (*p* < 0.001) together with a decrease of C18:1*n*-7 (*p* = 0.003) and C18:2*n*-6 (*p* = 0.008) fatty acids. No major changes were observed in terms of C20:4*n*-6, EPA and DHA.

### 2.3. Gene Expression Analysis

**Figure 1:**
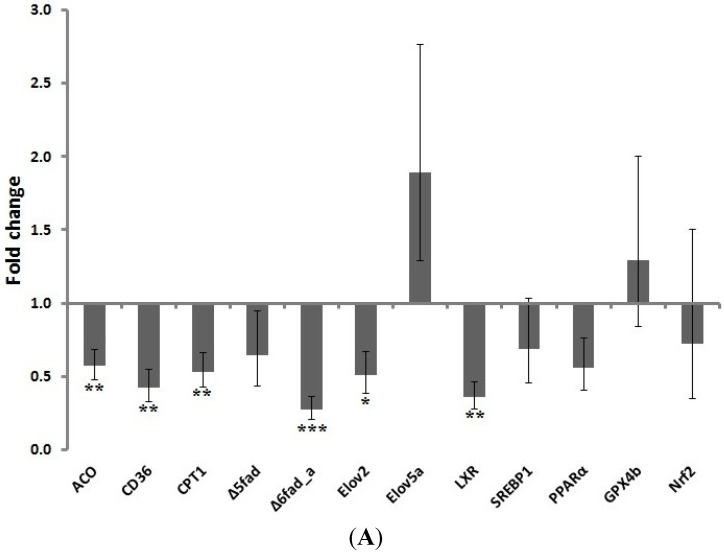

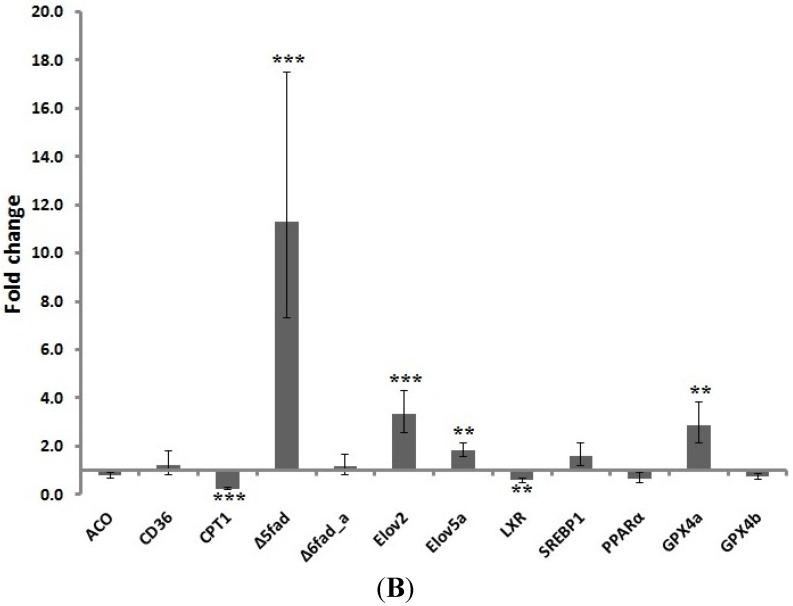
mRNA levels of genes encoding proteins centrally involved in fatty acid transport, synthesis and metabolism in salmon liver (**A**); and pyloric caeca (**B**). Relative gene expression values are shown as the fold change of the gamma tocopherol (gT) diet relative to the control diet (C) which was set to be 1.0 (*n* = 8 fish per group). Bars indicate the 95% confidence interval (Fold change up-Fold change low). (* *p* < 0.05; ** *p* < 0.01; *** *p* < 0.001).

Different gene expression patterns were observed in salmon liver (Figure 1A) and pyloric caeca (Figure 1B). A general down-regulation of both long chain PUFA synthesis (Δ6Fad_a, Elov2) and lipid transport (CD36) as well as beta oxidation related genes (*CPT1* and *ACO*) was observed in the liver of fish fed the diets enriched with gT (Figure 1A). Additionally, hepatic *LXR* expression was depressed (*p* < 0.01) in fish fed the gT rich diets, with no effect on *PPARα*, *SERBP1* and *Nrf2* expression (Figure 1B).

Contrarily, in the pyloric caeca of fish fed gT there was an overall increase of genes encoding proteins involved in long chain PUFA synthesis (Δ5 Fad, Elov2 and Elov5a) (*p* < 0.01), and no response (CD36 and ACO) or a decrease (CPT1; *p* < 0.001) of lipid transport and beta oxidation related genes was evident (Figure 1B). As far as transcription factors are concerned, a significant down-regulation of LXR (*p* < 0.01) in gT fed fish was observed. Moreover, GPX4a expression was up-regulated (*p* < 0.01) due to gT in pyloric caeca (Figure 1B).

**Table 3:** Fatty acid composition (g/100 g total fatty acid) of fillet from Atlantic salmon fed either a control diet (C) or a diet enriched with gamma tocopherol (gT) ^a^.

Fatty Acid	C		gT		*p*
C14:0	1.54	±0.01	1.40	±0.01	0.08
C16:0	11.86	±0.05	11.67	±0.07	0.06
C18:0	3.06	±0.02	3.09	±0.05	0.70
∑SFA ^b^	18.24	±0.04	20.62	±0.12	0.10
C16:1*n*-7	1.86	±0.04	1.80	±0.01	0.17
C18:1*n*-9	44.59	±0.11	44.17	±0.16	0.06
C18:1*n*-7	1.87	±0.04	1.82	±0.06	0.53
C20:1*n*-9	2.79	±0.02	2.66	±0.04	0.01
∑MUFA ^c^	52.32	±0.11	51.74	±0.18	0.02
C18:2*n*-6	14.84	±0.02	14.77	±0.08	0.49
C20:2*n*-6	1.15	±0.01	1.05	±0.02	0.01
C20:4*n*-6	0.31	±0.01	0.33	±0.01	0.27
∑ (*n*-6) ^d^	16.82	±0.03	16.82	±0.07	0.99
C18:3*n*-3	5.09	±0.04	5.29	±0.06	0.02
C20:3*n*-3	0.75	±0.02	0.81	±0.03	0.12
C20:5*n*-3	1.55	±0.04	1.48	± 0.05	0.33
C22:5*n*-3	0.75	± 0.01	0.91	± 0.08	0.09
C22:6*n*-3	3.94	±0.08	4.13	±0.14	0.30
∑ (*n*-3) ^e^	13.50	±0.11	14.30	±0.25	0.01
*n*-3/*n*-6	0.80	±0.01	0.85	±0.01	0.01

^a^ Values are means ± SE (*n* = 8); ^b^ ∑SFA = sum of saturated fatty acids. Includes C14:0, C16:0, C17:0, C18:0 and C20:0; ^c^ ∑MUFA = sum of monounsaturated fatty acids. Includes C16:1*n*-9, C16:1*n*-7, C17:1, C18:1*n*-9, C18:1*n*-7, C20:1*n*-9 and C22:1 isomers; ^d^ ∑ (*n*-6) = sum of *n*-6 fatty acids. Includes C18:2, C18:3, C20:2, C20:4 and C22:4; ^e^ ∑ (*n*-3) = sum of *n*-3 fatty acids. Includes C18:3, C18:4, C20:3, C20:4, C20:5, C22:5 and C22:6.

**Table 4:** Fatty acid composition (g/100 g total fatty acid) of liver from Atlantic salmon fed either a control diet (C) or a diet enriched with gamma tocopherol (gT) ^a^.

Fatty Acid	C		gT		*p*
C14:0	0.90	±0.02	0.76	±0.04	0.01
C16:0	11.38	±0.55	12.06	±0.52	0.39
C18:0	5.64	±0.18	6.91	±0.23	0.0008
∑SFA ^b^	18.24	±0.55	20.62	±0.65	0.01
C16:1*n*-7	1.25	±0.06	1.03	±0.12	0.13
C18:1*n*-9	36.19	±1.47	33.29	±2.53	0.34
C18:1*n*-7	1.94	±0.03	1.70	±0.05	0.003
C20:1*n*-9	3.84	±0.19	3.59	±0.19	0.37
∑MUFA ^c^	44.18	±1.62	40.60	±2.81	0.28
C18:2*n*-6	10.18	±0.29	8.89	±0.30	0.008
C20:2*n*-6	2.06	±0.09	2.01	±0.13	0.75
C20:4*n*-6	1.81	±0.14	2.36	±0.31	0.13
∑ (*n*-6) ^d^	14.89	±0.19	13.57	±0.22	0.0005
C18:3*n*-3	2.15	±0.13	1.85	±0.09	0.08
C20:3*n*-3	2.41	±0.13	2.67	±0.20	0.3
C20:5*n*-3	2.67	±0.25	2.75	±0.32	0.85
C22:5*n*-3	1.12	±0.07	1.19	±0.15	0.65
C22:6*n*-3	13.03	±0.92	15.39	±1.69	0.24
∑ (*n*-3) ^e^	22.35	±1.22	24.89	±2.16	0.32
*n*-3/*n*-6	1.50	±0.08	1.83	±0.14	0.08

^a^ Values are means ± SE (*n* = 8); ^b^ ∑SFA = sum of saturated fatty acids. Includes C14:0, C16:0, C17:0, C18:0 and C20:0; ^c^ ∑MUFA = sum of monounsaturated fatty acids. Includes C16:1*n*-9, C16:1*n*-7, C17:1, C18:1*n*-9, C18:1*n*-7, C20:1*n*-9 and C22:1 isomers; ^d^ ∑ (*n*-6) = sum of *n*-6 fatty acids. Includes C18:2, C18:3, C20:2, C20:4 and C22:4; ^e^ ∑ (*n*-3) = sum of *n*-3 fatty acids. Includes C18:3, C18:4, C20:3, C20:4, C20:5, C22:5 and C22:6.

## 3. Discussion

Marine functional foods may supply the consumer with macro- and micronutrients as well as nutraceuticals, thereby positively affecting cardiovascular health. In general, seafood and especially oily fish, such as Atlantic salmon, seem to be attractive sources to deliver fat soluble vitamins such as tocopherols since Vitamin E absorption is positively correlated with the lipid level of the diet. Furthermore, it has been suggested that tocopherols consumed within a complex food matrix may prevent cardiovascular disease more efficiently as compared to purified Vitamin E supplements [18].

Our data indicate that feeding Atlantic salmon with gT-rich diets resulted in a significant increase in gT concentrations in fish fillet, thereby improving its nutritional value. Thus, Atlantic salmon could be considered as a potential marine functional source of gT for human nutrition. However, future human trials (e.g., bioavailability studies, intervention studies) are required to test the hypothesis whether the consumption of gT-rich Atlantic salmon results in increased plasma levels of gT that may lead to potential anti-inflammatory and anti-atherogenic effects.

Interestingly, high dietary gT concentrations did not impair alpha tocopherol tissue levels. Thus, under the conditions investigated, no antagonistic interaction between gamma and alpha tocopherol was evident. Contrarily, high dietary alpha tocopherol concentrations have been reported to decrease gT tissue concentrations [5]. This is possibly related to the induction of cytochrome P450 enzymes which are involved in the metabolism of tocopherols and preferentially degrade the desmethyl vitamers [19].

Furthermore, we observed lower hepatic catalase and superoxide dismutase activities which may be indicative of an overall lowered oxidative stress level in the gT-fed salmon. Dietary gT supplementation significantly decreased MDA concentration in fish fillet. MDA is a biomarker of lipid peroxidation and impairs the sensory quality of oily fish [17]. Current as well as literature data [20] suggest that dietary gT could possibly improve the sensory quality of oily fish by reducing lipid peroxidation. In this context, future studies are needed to evaluate whether gT affects the shelf life of Atlantic salmon.

The enrichment of fat soluble pro-vitamins and vitamins into the food matrix is a common practice in aquaculture. However, the transfer of micronutrients including Vitamin E from the diet into the animal tissue is always accompanied by a loss in assimilation. In order to quantify the apparent Vitamin E absorption, digestibility experiments need to be conducted which was beyond the scope of our study. Furthermore, Vitamin E transfer may be experimentally determined by its whole body retention. In order to do so, Vitamin E levels need to be quantified in all tissues at the beginning and the end of a feeding trial. However, within this study Vitamin E was only determined in fillet and liver at the end of the study. Thus, it was not possible to pinpoint the exact Vitamin E retention. However, analyzed alpha and gamma tocopherol levels in liver and fillet suggest that the tissue retention of alpha tocopherol was higher as compared to gamma tocopherol which is in line with literature data [14]. In terms of alpha tocopherol, it has been suggested that around 40% of dietary alpha tocopherol is retained in the fillet of Atlantic salmon [14]. Since in the fillet of our salmon following dietary supplementation, the gamma tocopherol concentration was 2.8 fold lower as compared to alpha tocopherol a transfer efficiency of around 15% may be assumed.

Irrespective of a loss of retention, our experimental approach leads to a marine product rich in both, omega-3 fatty acids as well as alpha and gamma tocopherol. In this context it has been suggested that the consumption of 200 g Atlantic salmon would be sufficient to fulfill roughly 30% of the dietary Vitamin E recommendation [5]. Furthermore, it should be taken into account that oily fish does not only provide omega 3 fatty acids but is also an important source of protein, minerals and trace elements.

Besides its antioxidant activity, Vitamin E also plays an important role in signal transduction [21]. Both isoforms alpha and gamma tocopherol seem to affect lipid homeostasis in different ways. Studies in hepatocytes and Caco-2 cells have shown a decrease in cholesterol synthesis and secretion due to tocopherols via down-regulation of LXR, BCA1, ABCG1, SREBP1c or SREBP-2 transcription factors [22,23]. Additionally, there is evidence that alpha tocopherol can modulate the expression and activity of Δ9 and Δ6 fad [22,24]. In the present study, salmon fed the gT-enriched diets exhibited a significant hepatic down-regulation of different genes encoding proteins involved in lipid transport (CD36) and beta oxidation (CPT1 and ACO) as well as long chain PUFA synthesis (Δ6fad_a and elov2) which was mediated by decreased *LXR* gene expression. Together with the observed trend towards an increase of C18:3*n*-3 in the fillet, the decrease in liver C18:1*n*-7, C18:2*n*-6 and the accumulation of C18:0 may be the result of an overall down-regulation of lipid transport and beta oxidation as well as a decreased elongation and desaturation of C18:0. There are several molecular mechanisms by which tocopherol or its metabolites may affect gene expression and enzyme function [21,25]. The structural changes that tocopherol promotes when it is embedded in the membrane can affect lipid homeostasis [21]. Moreover, gT exerts different structural influence on the membrane than alpha tocopherol [26]. Studies in fish also suggest an indirect effect of Vitamin E on long chain PUFA synthesis by altering the cellular redox status [27]. Therefore, the efficient accumulation of both alpha and gamma tocopherol in salmon liver and lowered oxidative stress levels might have contributed to the changes observed in gene expression.

Similarly, CPT1 and transcription factor gene expression was down-regulated in the pyloric caeca of salmon fed gT. However, *Δ5 fad*, *Elov2* and *Elov5a* expression was increased. Different gene expression patterns of elongases and desaturases have been reported in the liver and pyloric caeca of salmon fed vegetable oils [28,29]. Additionally, the expression of the antioxidant enzyme GPX4a, which reduces phospholipid hydroperoxides [30], in the pyloric caeca of fish fed gT suggests a protective role of gT in lipid peroxidation. Moreover, Malandrakis *et al.* [31] suggested that the GPx status, which was improved by gamma tocopherol in our study, might be considered as an important indicator of fish welfare.

## 4. Experimental Section

### 4.1. Animals and Diets

A feeding trial with 120 Atlantic salmon post-smolt was performed at the Skretting Aquaculture Research Center (ARC) fish trial station (Lerang Research Station, Jorpeland, Norway). All experimental procedures were performed according to the Norwegian Animal Research Authority (FDU) guidelines. Fish with an initial average weight of 137.4 ± 1 g were randomly assigned to four 1 m deep × 1 m diameter circular tanks supplied with sea water and fed one of the two experimental diets for 16 weeks. Water temperature over the experimental period averaged 12.2 ± 0.2 °C. A basal control diet (C) was formulated to meet salmon nutrient recommendations [32] by using low fishmeal and fish oil concentrations (Table 5). The gamma tocopherol diet (gT) was the basal diet supplemented with 0.6 g/kg of d-γ-tocopherol (Chromadex). The analyzed composition of gT was 15 ppm and 170 ppm for the C and the gT diets, respectively. The dietary treatments were prepared at Skretting ARC pilot plant (Stavanger, Norway), as extruded, sinking 4 mm pellets.

### 4.2. Sampling

At the beginning, after formalin treatment and at the end of the experiment, all fish were individually weighed and measured for growth monitoring. For the final sampling, fish were deprived of feed for 12 h and then anesthetized and killed by a blow to the head. Four fish per tank (*n* = 8 per·diet) were selected to obtain individual samples of liver, fillet and plasma. Tissues were collected, snap frozen and stored at −80 °C for fatty acid, enzyme, tocopherol and MDA analyses. Approximately, 1 g of liver and mucosal scrapings from pyloric caeca were placed in RNA later and stored at −80 °C for gene expression analysis.

**Table 5:** Ingredients and analyzed chemical composition of the basal diet.

**Ingredients**	**As Fed Basis (g/kg)**
Wheat	50.0
Wheat gluten	168.1
Faba beans dehulled	93.8
Soy protein concentrate	310.0
Fishmeal NA	100.0
Palm oil	27.1
Linseed oil	11.7
Rapeseed oil	163.0
Fish oil NA	38.9
Astaxanthin 10%	0.4
Vitamin and mineral mix	37.1
**Analyzed composition**	
Moisture, %	6.6
Total fat, %	26.7
Crude protein, %	45.6
Ash, %	4.6
α-tocopherol (ppm)	192
γ-tocopherol (ppm)	15
**Fatty acids**	**Total fatty acids (g/100 g)**
C16:0	10.35
∑SFA ^a^	15.27
C18:1*n*-9	41.17
∑MUFA ^b^	49.10
C18:2*n*-6	17.39
∑ (*n*-6)	18.03
C18:3*n*-3	7.61
C20:5*n*-3	1.93
C22:6*n*-3	1.87
∑ (*n*-3)	12.45
*n*-3/*n*-6	0.69

^a^ ∑SFA = sum of saturated fatty acids; ^b^ ∑MUFA = sum of monounsaturated fatty acids.

### 4.3. Chemical and Enzyme Analysis

Feeds were analyzed for moisture, total fat, protein and ash by using in-house near-infrared reflectance (NIR) methodology at the Skretting ARC laboratory as previously described by [33]. Fatty acid analysis of the experimental diets was conducted by using gas chromatography and flame ionization detection [34]. Alpha and gamma tocopherol isomers of the experimental diets were analyzed using HPLC according to method [35]. Alpha and gamma tocopherols in salmon fillets and liver were extracted and quantified according to Faizan *et al.* [5]. Briefly, the tissues were homogenized in ethanol with ascorbic acid and KOH. After saponification, butylhydroxytoluol in ethanol and *n*-hexane were added and the organic phase was dried and dissolved in methanol. The separation of alpha and gamma tocopherol was performed by HPLC (Jasco GmbH Deutschland, Gross-Umstadt, Germany) on a Waters Spherisorb ODS-2 column (100 mm × 4.6 mm; 3 μm) using methanol/water (98:2, v/v) as mobile phase. The fluorescence detector was set to an excitation wavelength of 296 nm and emission wavelength of 325 nm.

Tissue lipids were extracted with dichloromethane-methanol (8:2) and fatty acids were methylated in the presence of sulphuric acid following the method of Segura and López-Bote [36]. Fatty acid methyl esters were then analyzed using a gas chromatograph (HP 6890 Series GC System) equipped with a flame ionization detector. Lipid peroxidation products in salmon fillets were determined as MDA concentrations by HPLC analysis according to Faizan *et al.* [17]. Briefly, fish tissue was homogenized in 1% sulfuric acid, addition of 0.2 volumes 6M NaOH was followed by a 30 min incubation at 60 °C, proteins were precipitated by adding perchloric acid and after centrifugation the supernatant was incubated with 2,4**-**dinitrophenylhydrazine and injected into a Jasco HPLC system. Using an isocratic mode with a mobile phase consisting of 0.2% acetic acid (v/v) in water/acetonitrile (42:58, v/v) the samples were separated on a Supelco INC water spherisorb ODS2 column (10 cm × 4.6 mm, 3 μm). MDA was analyzed at 310 nm.

Glutathione (GSH) was detected according to a modified method described previously [37]. Briefly, tissues were homogenized in 0.25 M sucrose, 3 mM EDTA, 10 mM Tris buffer pH 7.4 in bidest. water, Immediately after homogenization, the samples were mixed with equal amounts of ice-cold 10% (w/v) meta-phosphoric acid, incubated on ice and centrifuged before the clear supernatants were stored until analysis or analyzed directly by HPLC with a Kinetex C18 column (100 × 4.6 mm, 2.6 μm; Phenomenex, Aschaffenburg, Germany) and a 50 mM NaH_2_PO_4_ (adjusted to pH 3.0 with orthophosphoric acid) mobile phase. HPLC-EC detection of GSH was performed using an ESA (Chelmsford, MA, USA) colorimetric detector [38].

Hepatic superoxide dismutase was determined according to Marklund and Marklund [39] by measuring the inhibition of pyrogallol auto-oxidation spectrophotometrically. Catalase activity was measured according to Johansson and Borg [40] by measuring the absorption at 540 nm after adding methanol, hydrogen peroxide and purbald to the samples. For both assays, the supernatants from centrifuged tissues that had been homogenized in ice-cold PBS were used.

### 4.4. Gene Expression Analysis

Salmon liver and pyloric caecum RNA extraction and quality analysis was performed as indicated by Ipharraguerre *et al.* [41]. Reverse transcription was performed using the SuperScript VILO Master Mix (Invitrogen, Carlsbad, CA, USA) following the manufacturer’s guidelines. qRT-PCR was carried out using the SYBR^®^ Green Master Mix (Applied Biosystems, Foster City, CA, USA) in a 7300 Real Time PCR System (Applied Biosystems, Foster City, CA, USA). Primers and PCR conditions for salmon housekeeping genes β-actin and eukaryotic translation elongation factor 1 alpha (EF-1α) as well as Δ6 fad, carnitine palmitoyltransferase 1 (CPT1), and acyl CoA oxidase (ACO) were assayed as described by Leaver *et al.* [42], Δ5 fad and elongase 2 (Elov2) according to Morais *et al.* [28], Elov5a and LXR according to Minghetti *et al.* [43], CD36 according to Schiller *et al.* [44] and glutathione peroxidase 4a and b (GPX4a and GPX4b) according to Wang *et al.* [30] (Table 6).

Primers for the expression analysis of sterol receptor binding protein 1 (SREBP1), nuclear factor-like 2 (Nrf2), and PPARα were designed from sequences NM_001195818, BT059007.1 and AM230809.1, respectively, using Primer Express Software (Applied Biosystems, CA, USA; Table 2). The concentration and annealing temperatures for SREBP1 were 0.2 μM and 60 °C; 0.4 µM and 62 °C for PPARα; and 0.2 μM and 60 °C for Nrf2.

### 4.5. Statistical Analysis

Differences between means induced by gT relative to the C diet were detected through a *t*-test analysis using the PROC TTEST from SAS (release 9.2, SAS Institute Inc., Cary, NC, USA) [45]. For genes displaying efficiencies different from 2 (E ≠ 2), Ct values were adjusted according to the model described by Steibel *et al.* [46]. The geometric mean of the reference genes β-actin and EF-1α was used to correct Ct values of target genes [47].

**Table 6:** Genes and forward (Fw) and reverse (Rv) primers used for gene expression analysis by qRT-PCR.

Genes	Primer Sequence Fw	Primer Sequence Rv	Reference
*Δ6fad_a*	CCCCAGACGTTTGTGTCAG	CCTGGATTGTTGCTTTGGAT	[42]
*ACO*	AAAGCCTTCACCACATGGAC	TAGGACACGATGCCACTCAG	[42]
*β-actin*	ACATCAAGGAGAAGCTGTGC	GACAACGGAACCTCTCGTTA	[42]
*EF-1α*	CTGCCCCTCCAGGACGTTTCAA	CACCGGGCATAGCCGATTCC	[42]
*Δ5 fad*	GTGAATGGGGATCCATAGCA	AAACGAACGGACAACCAGA	[28]
*Elov2*	CGGTACAAAATGTGCTGGT	TCTGTTTGCCGATAGCCATT	[28]
*Elov5a*	ACAAGACAGGAATCTCTTTCAGATTAA	TCTGGGGTTACTGTGCTATAGTGTAC	[43]
*LXR*	GCCGCCGCTATCTGAAATCTG	CAATCCGGCACCAATCTGTAGG	[43]
*CD36*	GGATGAACTCCCTGCATGTGA	TGAGGCCAAAGTACTCGTCGA	[44]
*SREBP1*	CACTACTAGCCCCATGTTTTGATTG	CAGCCACTCTCTAAACACACCAA	
*PPARα*	GCTCCTTGGATGTCCCTGAGT	GCATCTAGAACGGTGGATCCTT	
*Nrf2*	GGTTTCCAGACTTCTCTCTCAGTGT	GAACATGGCAAGACCGAGCC	
*GPX4a*	GTACGCTGAGAAAGGTTTACGC	TTGATGCCATTTCCCAGG	[30]
*GPX4b*	ATCACCAACGTTGCCTCTAAAT	CCTTGATTTCCACCTCTGTACC	[30]
*CPT1*	CCTGTACCGTGGAGACCTGT	CAGCACCTCTTTGAGGAAGG	[42]

## 5. Conclusions

The present findings suggest that salmon fillet can be enriched with gT when provided in the feed. Moreover, no antagonistic interaction between gamma and alpha tocopherol was observed in salmon tissues after high dietary gT feeding. This seems to improve both the nutritional value, with higher tocopherol and a slight increase of *n*-3 concentrations, and the sensory quality of Atlantic salmon. Moreover, gT increases fish GPx status and hence its welfare. Overall, Atlantic salmon may be an important marine functional source capable of improving gT supply in humans.
